# Design and Self-Assembling Behaviour of Calamitic Reactive Mesogens with Lateral Methyl and Methoxy Substituents and Vinyl Terminal Group

**DOI:** 10.3390/polym13132156

**Published:** 2021-06-30

**Authors:** Alexej Bubnov, Martin Cigl, Deyvid Penkov, Marek Otruba, Damian Pociecha, Hsiu-Hui Chen, Věra Hamplová

**Affiliations:** 1 Institute of Physics of the Czech Academy of Sciences, Na Slovance 1999/2, 182 21 Prague, Czech Republic; cigl@fzu.cz (M.C.); hamplova@fzu.cz (V.H.); 2 Gymnázium Christiana Dopplera, Zborovská 621/45, 150 00 Prague, Czech Republic; penkover@seznam.cz (D.P.); marekpopovicky@seznam.cz (M.O.); 3 Faculty of Chemistry, University of Warsaw, ul. Zwirki i Wigury 101, 02-089 Warsaw, Poland; pociu@chem.uw.edu.pl; 4 Institute of Organic and Polymeric Materials, Research and Development Center of Smart Textile Technology, National Taipei University of Technology, Taipei 106, Taiwan; hhchen@ntut.edu.tw

**Keywords:** liquid crystal, vinyl group, reactive mesogen, smectic phases, self-assembling behaviour

## Abstract

Smart self-organising systems attract considerable attention in the scientific community. In order to control and stabilise the liquid crystalline behaviour, and hence the self-organisation, the polymerisation process can be effectively used. Mesogenic units incorporated into the backbones as functional side chains of weakly cross-linked macromolecules can become orientationally ordered. Several new calamitic reactive mesogens possessing the vinyl terminal group with varying flexible chain lengths and with/without lateral substitution by the methyl (methoxy) groups have been designed and studied. Depending on the molecular structure, namely, the type and position of the lateral substituents, the resulting materials form the nematic, the orthogonal SmA and the tilted SmC phases in a reasonably broad temperature range, and the structure of the mesophases was confirmed by X-ray diffraction experiments. The main objective of this work is to contribute to better understanding of the molecular structure–mesomorphic property relationship for new functional reactive mesogens, aiming at further design of smart self-assembling macromolecular materials for novel sensor systems.

## 1. Introduction

Functional self-assembling systems’ build-up from the organic molecules attract considerable attention from the scientific community due to their extraordinary properties that can be utilised for smart applications [1,2,3]. Some soft organic materials with definite molecular structure can exhibit the liquid crystalline (LC), i.e., self-assembling, behaviour, that can be changed, tuned and controlled by an external stimulus: applied electric/magnetic field, mechanical stress and irradiation by UV or visible light [3,4,5]. A tremendous number of materials, mixtures and composites possessing the LC behaviour have been designed and investigated during the last decades, many of which already respond to the demands of specific applications, such as various display and opto-electronic devices. Nevertheless, the information gained so far is still insufficient for the effective applicability of LC materials for further smart applications, the reason being that the control and prediction of the LC properties requires further efforts to assure the stability of the target LC mesophases and other specific properties.

In order to stabilise the liquid crystalline behaviour, and hence the self-organisation, the polymerisation process can be effectively used [6,7,8,9]. Mesogenic units, also called reactive mesogens, incorporated into the polymeric backbones as side chains of weakly cross-linked macromolecules, can form orientationally ordered nanostructures. The resultant material may combine the anisotropy and large susceptibility of low molar-mass LCs with mechanical elasticity and relatively simple processability of macromolecular materials. During recent decades, an increased interest in advanced macromolecular materials possessing certain properties [4,10,11,12,13], specifically in LC side-chain polymers and elastomers possessing the self-assembling behaviour, has occurred due to their high potential for various smart applications, such as artificial muscles, micro-valves, mechanical actuators, smart surfaces and propulsion systems, etc. [5,14]. The self-organisation behaviour build-up on the intermolecular interactions can be effectively tuned by the appropriate molecular design, e.g., by constructing the macromolecular system from the mesogenic units with the reactive terminal groups [15,16,17]. However, it is very difficult to theoretically predict, describe and keep under control the macroscopic parameters of the self-assembling materials [18,19,20]. Many experimental efforts in reactive mesogen design [4] have been carried out while building-up new photosensitive and photo-controllable macromolecular materials [10], incorporating a photo-responsive azo group into the molecular core. Its presence allows the effective driving of the resulting optical, structural, mesomorphic and dielectric properties by non-contact stimuli, i.e., irradiation by UV or even by visible light [16,21,22,23].

Utilisation of the various types of polymeric backbones (polyacrylate [16], polymethacrylate [4,21,22,24], polysiloxane [23,25,26,27,28], polyurethane [29], etc.) for the design of LC macromolecular materials requires a specific molecular structure of the reactive terminal groups (acrylate [16,30,31,32,33], methacrylate [4,21,22,32], vinyl [12,23,27,34], thiols [35,36,37], etc.) of the respective monomeric LC materials used as functional reactive mesogens. However, each part of the reactive mesogen molecule, namely the molecular core, type and position of the lateral substitution, linkage groups, length and type of the flexible chains, etc., can significantly change the resulting self-assembling behaviour of the designed material. The methyl [10,22,38,39] and methoxy [39,40,41] groups and halogen atoms [38,42], namely fluorine [42,43,44,45,46,47], chlorine [38,41,42,46,48,49], bromine [38,43] and iodine [50], are the most widely used and successful types of lateral substituents utilised for the effective tuning of the self-assembling behaviour, especially for the non-chiral and chiral calamitic LC materials. The type and position of the lateral substituents on the molecular core always drastically change the liquid crystalline behaviour of the resulting LC material when compared with its non-substituted analogue [38,39]. Specifically, it has been shown [38,39] that the lateral substitution on the phenyl ring close to the terminal chain can induce the nematic (or the cholesteric) phase, while for the non-substituted materials, the smectic phases are more favourable [38,39]. The type and position of the lateral substitution considerably affects the thermal properties of the materials, i.e., the existence and the temperature range of the mesophases [38,39,40].

The main objective of our research is to contribute to better understanding of the molecular architecture—self-assembling behaviour relationship for the functional reactive mesogens, aiming at further design of smart self-organising macromolecular materials, such as the LC side-chain polymers and single LC elastomers, targeted for novel sensor systems.

**Figure 1:**
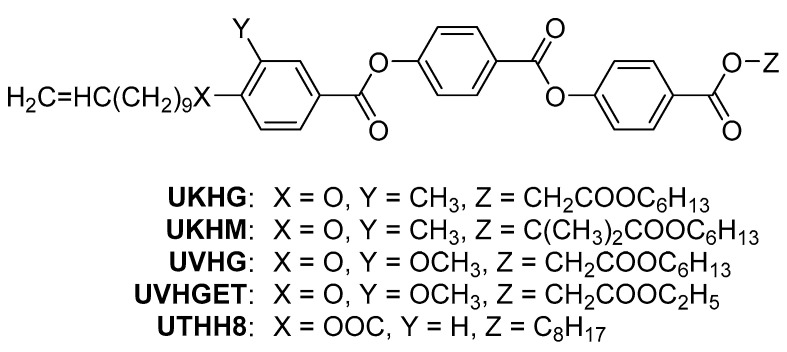
General chemical structure of the designed laterally substituted reactive mesogens with vinyl terminal group.

Specifically, we aim to check the effect of the lateral substitution by the methyl and methoxy groups on the self-assembling behaviour for calamitic reactive mesogens with vinyl terminal group. In order to realise the objective, several new calamitic reactive mesogens (see Figure 1) with/without lateral substitution by the methyl/methoxy groups possessing different lengths of flexible terminal chains have been designed and studied.

## 2. Materials and Methods

This section contains a detailed description of the synthesis and confirmation of the molecular structures obtained for all of the designed reactive mesogens. Additionally, it provides the description of the experimental techniques used for the characterisation of the mesomorphic, thermal and structural properties.

### 2.1. Design and Synthesis

All starting materials and reagents were purchased from local ditributors of Sigma-Aldrich (Merck), Acros Organics or Fluorochem. Solvents used for the syntheses were “p.a.” purity grade. ^1^H NMR spectra were recorded on Varian VNMRS 300; deuteriochloroform (CDCl_3_) was used as solvent and the signal of solvent served as the internal standard. Chemical shifts (δ) are given in ppm and spin-spin coupling constants (*J*) are given in Hz. Column chromatography was carried out using Merck Kieselgel 60 (60−100 μm). The purity of final compounds was checked by HPLC analysis (high-pressure pump ECOM Alpha; column WATREX Biospher Si 100, 250 × 4 mm, 5 μm; detector WATREX UVD 250) and were found to be >99.8%. 

**Figure 2:**
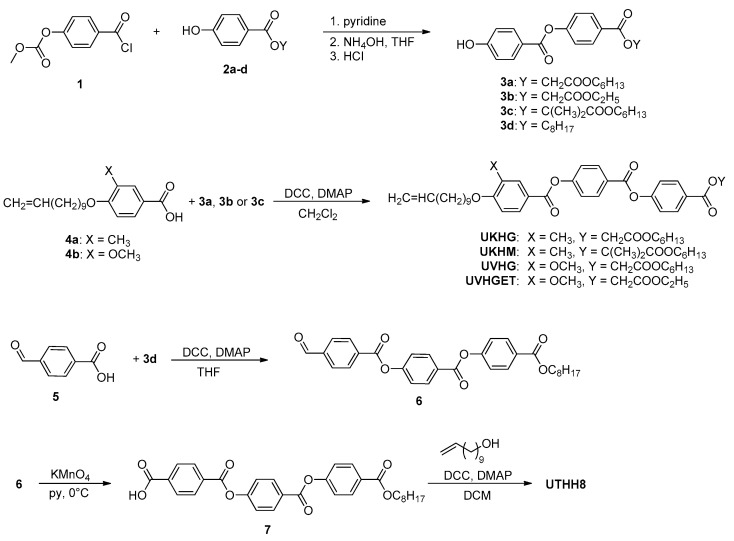
General scheme for synthesis of new reactive mesogens with vinyl terminal group possessing the lateral substitution on the molecular core by the methyl and methoxy groups.

Newly designed reactive mesogens have been synthesised according to the synthetic route described in Figure 2. First, benzoyl chloride **1** and hydroxy-esters **2a**–**d** were synthesised following the procedures from the literature [17]. The reaction of benzoyl chloride **1** with appropriate hydroxy-ester **2a**–**d** and the subsequent deprotection of hydroxyl by means of aqueous ammonia yielded hydroxy-esters **3a**–**d**. In the next step, acids **4a** and **4b**, which were synthesised as recently described [17], were reacted with hydroxy-esters **3a–c** in a DCC-mediated reaction, resulting in reactive mesogens denoted as UKHG, UKHM, UVHG and UVHGET (see Figure 2).

Synthesis of UTHH8 reactive mesogen was started from hydroxyester **3d**, which was originally esterified with 4-formylbenzoic acid (**5**). Aldehyde **6** was then oxidized to benzoic acid **7** using potassium permanganate in pyridine. In the final step, acid **7** was esterified with 10-undecenol by means of DCC coupling.

*4′-{[2-(Hexyloxy)-2-oxoethoxy]carbonyl}phenyl 4-hydroxybenzoate* (**3a**)

Benzoyl chloride **1** (4.0 g, 18.64 mmol) dissolved in toluene (20 mL) was added dropwise to the stirred mixture of **2a** (5.20 g, 18.55 mmol) and dry pyridine (8 mL) in toluene (70 mL). The reaction mixture was stirred for 6 h and then refluxed for 30 min. The resulting cooled mixture was filtered, and the filtrate was washed with diluted hydrochloric acid (100 mL, 5%) and water (100 mL). The separated organic layer was dried with anhydrous magnesium sulphate. After the evaporation of the solvent, the residue was dissolved in tetrahydrofuran (50 mL) and cooled to −20 °C. To this precooled solution, concentrated aqueous ammonia (10 mL, 25%, 64.19 mmol) was added, with constant stirring. The reaction mixture was stirred and let warm to room temperature, and the progress of the hydrolysis was monitored by TLC. After ca. 45 min, the resulting mixture was poured into water (100 mL) and neutralised with hydrochloric acid. The organic layer was separated, and the aqueous layer was extracted with diethylether (2 × 50 mL). Combined organic layers were washed with water (50 mL) and dried over anhydrous sodium sulphate. Solvent was removed under reduced pressure and the oily residue was purified by chromatography on silica (dichloromethane:acetone (96:4)). Yield = 5.96 g (80%) of viscous liquid **3a**.

^1^H NMR (CDCl_3_): 8.12 (2 H, d, *J =* 8.8, H-3′, H-5′), 8.02 (1 H, d, *J =* 8.7, H-2, H-6), 7.28 (2 H, d, *J =* 8.8, H-2′, H-6′), 6.90 (2 H, d, *J =* 8.7, H-3, H-5), 4.88 (2 H, s, CH_2_COO), 4.22 (2 H, t, *J =* 6.4, COOCH_2_), 1.77–1.94 (2 H, m, C**H**_2_CH_2_O), 1.60–1.73 (2 H, m, C**H**_2_CH_2_O), 1.21–1.35 (6 H, m, 3 × CH_2_), 0.88 (3 H, *J* = 6.7, CH_2_C**H**_3_).

*4′-{[2-(Ethoxy)-2-oxoethoxy]carbonyl}phenyl 4-hydroxybenzoate* (**3b**)

Using the procedure described for **3a**, starting from benzoyl chloride **1** (1.0 g, 4.66 mmol) and hydroxy-ester **2b** (1.0 g, 4.46 mmol), and the subsequent hydrolysis of the carbonate protective group, 1.35 g (88%) of hydroxy-ester **3b** was obtained.

^1^H NMR (CDCl_3_) 8.12 (2 H, d, *J =* 8.8, H-3′, H-5′), 8.02 (1 H, d, *J =* 8.7, H-2, H-6), 7.28 (2 H, d, *J =* 8.8, H-2′, H-6′), 6.90 (2 H, d, *J =* 8.7, H-3, H-5), 4.88 (2 H, s, CH_2_COO), 4.28 (2 H, q, *J* = 7.2, **C**OOCH_2_), 1.36 (3 H, *J* = 6.7, CH_2_C**H**_3_).

*1-(Hexyloxy)-2-methyl-1-oxopropan-2-yl 4-[(4-hydroxybenzoyl)oxy]benzoate* (**3c**)

Starting from benzoyl chloride **1** (2.0 g, 9.32 mmol) and hydroxy-ester **2c** (2.87 g, 9.31 mmol), 3.10 g (78%) of hydroxy-ester **3c** was obtained by the procedure described for **3a**.

^1^H NMR (CDCl_3_) 8.12 (2 H, d, *J =* 8.8, H-3′, H-5′), 8.02 (1 H, d, *J =* 8.7, H-2, H-6), 7.28 (2 H, d, *J =* 8.8, H-2′, H-6′), 6.90 (2 H, d, *J =* 8.7, H-3, H-5), 4.15 (2 H, t, *J* = 6.7, COOC**H**_2_), 1.68 (6 H, s, (C**H**_3_)_2_C), 1.62 (2 H, quin. *J* = 7.0, COOCH_2_C**H**_2_), 1.11–1.45 (6 H, m, 3 × CH_2_), 0.89 (3 H, *J* = 6.7, CH_2_C**H**_3_).

*4′-[(Octyloxy)carbonyl]phenyl 4-hydroxybenzoate* (**3d**)

In the same procedure as that of **3a**, chloride **1** (1.0 g, 4.66 mmol) was reacted with hydroxy-ester **2d** (1.17 g, 4.67 mmol), and the subsequent hydrolysis of the carbonate protective group yielded 1.55 g (90%) of ester **3d**.

^1^H NMR (CDCl_3_) 8.10–8.22 (4 H, m, H-2, H-6, H-3′, H-5′), 7.28 (2 H, d, *J* = 8.5, H-2′, H-6′), 6.93 (2 H, d, *J =* 8.5, H-3, H-5), 4.32 (2 H, t, *J* = 6.7, COOC**H**_2_), 1.77 (2 H, quin. *J* = 7.0, COOCH_2_C**H**_2_), 1.10–1.55 (10 H, m, 5 × CH_2_), 0.87 (3 H, *J* = 6.7, CH_2_C**H**_3_).

*Octyl 4-({4′-[(4′′-formylbenzoyl)oxy]benzoyl}oxy)benzoate* (**6**)

A mixture of 4-formylbenzoic acid (1.50 g, 9.89 mmol) and hydroxy-ester **3d** (3.66 g, 9.88 mmol) was dissolved in tetrahydrofuran (50 mL) and cooled to 0 °C. Dicyclohexylcarbodiimide (DCC) (2.18 g, 10.35 mmol) and 4-(*N,N*-dimethylamino)pyridine (DMAP) (0.30 g, 2.46 mmol) were added, and the mixture was stirred for 3 h at room temperature. Precipitated *N,N’*-dicyclohexylurea was filtered off, and the resulting filtrate was washed with HCl (20 mL, 1:15) and water. The organic layer was dried with anhydrous sodium sulphate. Removal of the solvent under reduced pressure yielded benzoate **6** (4.72 g, 95%), which was utilised in the further step without additional purification.

^1^H NMR (CDCl_3_): 10.11 (s, 1H, CHO), 8.10–8.37 (m, 8 H, H-2, H-6, H-3′, H-5′, H-2′′, H-6′′, H-3′′, H-5′′), 7.44 (2 H, d, *J =* 8.8, H-2′, H-6′), 7.32 (2 H, d, *J =* 8.2, H-2, H-6), 4.33 (t, 2H, *J* = 6.7, OCH_2_), 1.74–1.88 (2 H, m, OCH_2_C**H**_2_), 1.16–1.60 (10 H, m, 5 × CH_2_), 0.88 (t, 3H, *J* = 6.8, CH_3_).

*4-{[4′-({4′′-[(Octyloxy)carbonyl]phenoxy}carbonyl)phenoxy]carbonyl}benzoic acid* (**7**)

The solution of potassium permanganate (1.55 g, 9.81 mmol) in water (20 mL) at ca. 50 °C was added in two portions with a 15 min interval to the agitated solution of 4-formylbenzoate **6** (4.70 g, 9.35 mmol) in pyridine (50 mL), cooled to −10 °C by the ice-salt bath. After the last addition, the mixture was kept overnight at −20 °C and then it was slowly added to the mixture of the concentrated HCl (50 mL) in the ice-cold water (100 mL). The resulting suspension was neutralised by an additional amount of the concentrated HCl (ca. 18 mL) and filtered through the suction with a pad of celite. Filtered solid was boiled with acetone (100 mL) and filtered again. Macerate was dried with anhydrous sodium sulphate. The evaporation of acetone yielded crude **7**, which was crystallised from hexane. Yield = 4.36 g (90%).

^1^H NMR (CDCl_3_): 8.10–8.35 (m, 8 H, H-2, H-6, H-3, H-5, H-3′, H-5′, H-3′′, H-5′′,), 7.44 (2 H, d, *J =* 8.8, H-2′, H-6′), 7.35 (2 H, d, *J =* 8.2, H-2′′, H-6′′), 4.36 (t, 2H, *J* = 6.7, OCH_2_), 1.70–1.84 (2 H, m, OCH_2_C**H**_2_), 1.19–1.65 (10 H, m, 5 × CH_2_), 0.88 (t, 3H, *J* = 6.8, CH_3_).

*4′-[(4′′-{[2-(Hexyloxy)-2-oxoethoxy]carbonyl}phenoxy)carbonyl]phenyl 3-methyl-4-(undec-10-en-1-yloxy)benzoate* (UKHG)

Ester **3a** (1.0 g, 3.28 mmol) and acid **4a** (1.31 g, 3.27 mmol) were dissolved in dry dichloromethane (50 mL) and cooled to 2–8 °C. Then, *N*,*N*′-dicyclohexylcarbodiimide (DCC) (0.71 g, 3.40 mmol) and 4-(*N,N*-dimethylamino)pyridine (DMAP) (0.1 g, 0.82 mmol) were added. The mixture was stirred for six hours and then filtered. The resulting filtrate was evaporated, and the residue was purified by column chromatography (silica gel, dichloromethane:acetone, 99.7:0.3) and recrystallised from hexane to obtain 2.04 g (91%) of UKHG.

^1^H NMR (CDCl_3_) 8.29 (2 H, d, *J =* 8.8, H-3′, H-5′), 8.20 (2 H, d, *J =* 8.8, H-3′′, H-5′′), 8.05 (1 H, dd, *J =* 8.8, 1.8, H-6), 8.00 (1 H, d, *J =* 1.8, H-2), 7.37 (4 H, dd, *J =* 11.7, 8.8, H-2′, H-6′, H-2′′, H-6′′), 6.90 (1 H, d, *J =* 8.8, H-5), 5.72–5.94 (2H, m, C**H**_2_=CH), 4.90–5.08 (1H, m, C**H**=CH_2_), 4.88 (2 H, s, CH_2_COO), 4.21 (2 H, t, *J =* 6.5, COOCH_2_), 4.07 (2 H, t, *J =* 6.5, OCH_2_), 2.30 (3 H, s, ArCH_3_), 1.97–2.15 (2 H, m, C**H**_2_CH=), 1.77–1.94 (2 H, m, C**H**_2_CH_2_O), 1.60–1.73 (2 H, m, C**H**_2_CH_2_O), 1.21–1.58 (18 H, m, 9 × CH_2_), 0.89 (3 H, *J* = 6.7, C**H**_3_CH_2_). ^13^C NMR (75 MHz, CDCl_3_): 167.83 (CH_2_**C**OO), 165.19 (**C**OOCH_2_), 164.54 (COOAr), 163.90 (C′OOAr), 162.01 (C-4), 155.68 (C-1′), 154.93 (C-1′′), 149.06 (C-3), 139.20 (**C**H=CH_2_), 132.56 (C-2), 131.88 (C-3′, C-5′), 131.62 (C-3′′, C-5′′), 130.27 (C-6), 127.19 (C-3), 126.87 (C-4′′), 126.25 (C-4′), 122.25 (C-2′, C-6′), 121.88 (C-2′′, C-6′′), 120.15 (C-1), 114.14 (CH=**C**H_2_), 110.18 (C-5), 68.27 (CH_2_OAr), 65.65 (COO**C**H_2_), 61.28 (**C**H_2_COO), 33.80 (**C**H_2_CH=), 31.33 (**C**H_2_CH_2_CH_3_), 29.02–29.57 (m, 5 × CH_2_), 28.91 (**C**H_2_CH_2_O), 28.44 (**C**H_2_CH_2_O), 26.04 (**C**H_2_(CH_2_)_2_O), 25.43 (**C**H_2_(CH_2_)_2_O), 22.50 (**C**H_2_CH_3_), 16.24 (ArCH_3_), 13.98 (CH_2_**C**H_3_). Elemental Analysis for C_41_H_50_O_9_ (686.83): calc. C 71.70, H 7.34; found C 71.54, H 7.20.

*4′-[(4′′-{[2-(Hexyloxy)-2-oxoethoxy]carbonyl}phenoxy)carbonyl]phenyl 3-methoxy-4-(undec-10-en-1-yloxy)benzoate* (UVHG)

Analogously to the case of UKHG (see above), acid **4b** (1.0 g, 3.12 mmol) was reacted with ester **3a** (1.25 g, 3.12 mmol) in the presence of DCC (0.68 g, 3.28 mmol) and DMAP (80.0 mg, 0.65 mmol) in dichloromethane. Yield = 1.91 g (87%).

^1^H NMR (CDCl_3_) 8.30 (2 H, d, *J =* 8.8, H-3′, H-5′), 8.21 (2 H, d, *J =* 8.8, H-3′′, H-5′′), 7.87 (1 H, dd, *J =* 8.5, 2.2, H-6), 7.68 (1 H, d, *J =* 2.2, H-2), 7.37 (4 H, dd, *J =* 12.7, 8.8, H-2′, H-6′, H-2′′, H-6′′), 6.97 (1 H, d, *J =* 8.5, H-5), 5.73–5.95 (2H, m, C**H**_2_=CH), 4.90–5.06 (1H, m, C**H**=CH_2_), 4.88 (2 H, s, CH_2_COO), 4.21 (2 H, t, *J =* 6.5, COOCH_2_), 4.13 (2 H, t, *J =* 6.5, OCH_2_), 3.97 (3 H, s, ArOCH_3_), 1.99–2.13 (2 H, m, C**H**_2_CH=), 1.84–1.98 (2 H, m, C**H**_2_CH_2_O), 1.61–1.75 (2 H, m, C**H**_2_CH_2_O), 1.21–1.58 (18 H, m, 9 × CH_2_), 0.89 (3 H, *J* = 6.7, C**H**_3_CH_2_). ^13^C NMR (75 MHz, CDCl_3_): 167.83 (CH_2_**C**OO), 165.17 (**C**OOCH_2_), 163.87 (COOAr), 163.06 (C′OOAr), 155.57 (C-1′), 154.90 (C-1′′), 153.56 (C-4), 149.06 (C-3), 139.20 (**C**H=CH_2_), 131.91 (C-3′, C-5′), 131.62 (C-3′′, C-5′′), 126.88 (C-4′′), 126.39 (C-4′), 124.60 (C-6), 122.24 (C-2′, C-6′), 121.87 (C-2′′, C-6′′), 120.84 (C-1), 114.14 (CH=**C**H_2_), 112.64 (C-2), 111.43 (C-5), 69.12 (CH_2_OAr), 65.65 (COO**C**H_2_), 61.28 (**C**H_2_COO), 56.16 (OCH_3_), 33.79 (**C**H_2_CH=), 31.33 (**C**H_2_CH_2_CH_3_), 29.02–29.63 (m, 5 × CH_2_), 28.93 (**C**H_2_CH_2_O), 28.44 (**C**H_2_CH_2_O), 25.87 (**C**H_2_(CH_2_)_2_O), 25.43 (**C**H_2_(CH_2_)_2_O), 22.50 (**C**H_2_CH_3_), 13.98 (CH_2_**C**H_3_). Elemental Analysis for C_41_H_50_O_10_ (702.83): calc. C 70.07, H 7.17; found C 71.17, H 7.25.

*4′-[(4′′-{[2-(Ethoxy)-2-oxoethoxy]carbonyl}phenoxy)carbonyl]phenyl 3-methoxy-4-(undec-10-en-1-yloxy)benzoate* (UVHGET)

Analogously to the case of UKHG, acid **4b** (1.0 g, 3.12 mmol) was reacted with ester **3b** (1.07 g, 3.11 mmol) in the presence of DCC (0.68 g, 3.28 mmol) and DMAP (80.0 mg, 0.65 mmol) in dichloromethane. Yield = 1.90 g (95%).

^1^H NMR (CDCl_3_) 8.30 (2 H, d, *J =* 8.8, H-3′, H-5′), 8.21 (2 H, d, *J =* 8.8, H-3′′, H-5′′), 7.86 (1 H, dd, *J =* 8.5, 2.1, H-6), 7.68 (1 H, d, *J =* 2.1, H-2), 7.37 (4 H, dd, *J =* 12.3, 8.8, H-2′, H-6′, H-2′′, H-6′′), 6.97 (1 H, d, *J =* 8.5, H-5), 5.70–5.92 (2H, m, C**H**_2_=CH), 4.90–5.07 (1H, m, C**H**=CH_2_), 4.87 (2 H, s, CH_2_**C**OO), 4.28 (2 H, q, *J* = 7.2, **C**OOCH_2_), 4.12 (2 H, t, *J =* 6.5, OCH_2_), 3.97 (3 H, s, ArOCH_3_), 1.99–2.11 (2 H, m, C**H**_2_CH=), 1.81–1.97 (2 H, m, C**H**_2_CH_2_O), 1.21–1.55 (15 H, m, 6 × CH_2,_ C**H**_3_CH_2_). ^13^C NMR (75 MHz, CDCl_3_): 167.73 (CH_2_**C**OO), 165.17 (**C**OOCH_2_), 164.39 (COOAr), 163.87 (C′OOAr), 155.57 (C-1′), 154.90 (C-1′′), 153.56 (C-4), 149.06 (C-3), 139.20 (**C**H=CH_2_), 131.91 (C-3′, C-5′), 131.64 (C-3′′, C-5′′), 126.87 (C-4′′), 126.39 (C-4′), 124.60 (C-6), 122.24 (C-2′, C-6′), 121.88 (C-2′′, C-6′′), 120.83 (C-1), 114.14 (CH=**C**H_2_), 112.64 (C-2), 111.41 (C-5), 69.10 (CH_2_OAr), 61.53 (**C**H_2_COO), 61.28 (COO**C**H_2_), 56.16 (OCH_3_), 33.79 (**C**H_2_CH=), 28.70–29.69 (m, 5 × CH_2_), 25.87 (CH_2_CH_2_O), 14.13 (CH_2_**C**H_3_). Elemental Analysis for C_37_H_42_O_10_ (646.72): calc. C 68.71, H 6.55; found C 68.59, H 6.66.

*4′-{[4′′-({[1-(Hexyloxy)-2-methyl-1-oxopropan-2-yl]oxy}carbonyl)phenoxy]carbonyl}phenyl 3-methyl-4-(undec-10-en-1-yloxy)benzoate* (UKHM)

Analogously to the case of UKHG, acid **4b** (1.0 g, 3.12 mmol) was reacted with ester **3c** (1.33 g, 3.10 mmol) in the presence of DCC (0.68 g, 3.28 mmol) and DMAP (80.0 mg, 0.65 mmol) in dichloromethane. Yield = 1.84 g (83%).

^1^H NMR (CDCl_3_) 8.29 (2 H, d, *J =* 8.8, H-3′, H-5′), 8.13 (2 H, d, *J =* 8.8, H-3′′, H-5′′), 8.05 (1 H, dd, *J =* 8.5, 1.8, H-6), 8.00 (1 H, d, *J =* 1.8, H-2), 7.39 (2 H, d, *J =* 8.8, H-2′, H-6′), 7.32 (2 H, d, *J =* 8.8, H-2′′, H-6′′), 6.90 (1 H, d, *J =* 8.5, H-5), 5.70–5.95 (2H, m, C**H**_2_=CH), 4.88–5.08 (1H, m, C**H**=CH_2_), 4.16 (2 H, t, *J =* 6.7, COOCH_2_), 4.07 (2 H, t, *J =* 6.5, OCH_2_), 2.30 (3 H, s, ArCH_3_), 1.98–2.13 (2 H, m, C**H**_2_CH=), 1.79–1.92 (2 H, m, C**H**_2_CH_2_O), 1.71 (6 H, s, C(CH_3_)_2_), 1.12–1.66 (18 H, m, 9 × CH_2_), 0.86 (3 H, *J* = 6.7, C**H**_3_CH_2_). ^13^C NMR (75 MHz, CDCl_3_): 172.61 (C**C**OO), 164.62 (COOAr), 163.92 (C′OOAr), 162.03 (C-4), 155.68 (C-1′), 154.93 (C-1′′), 149.06 (C-3), 139.20 (**C**H=CH_2_), 132.57 (C-2), 131.88 (C-3′, C-5′), 131.39 (C-3′′, C-5′′), 130.28 (C-6), 127.80 (C-3), 127.20 (C-4′′), 126.25 (C-4′), 122.25 (C-2′, C-6′), 121.73 (C-2′′, C-6′′), 120.16 (C-1), 114.15 (CH=**C**H_2_), 110.20 (C-5), 78.96 (**C**COO), 68.27 (CH_2_OAr), 65.53 (COO**C**H_2_), 33.79 (s,**C**H_2_CH=CH_2_), 31.31 (**C**H_2_CH_2_CH_3_), 28.97–29.65 (m, 5 × CH_2_), 28.92 (**C**H_2_CH_2_O), 28.37 (**C**H_2_CH_2_O), 26.06 (**C**H_2_(CH_2_)_2_O), 25.47 (**C**H_2_(CH_2_)_2_O), 24.74 (**C**(CH_3_)_2_), 22.47 (**C**H_2_CH_3_), 16.26 (ArCH_3_), 13.97 (CH_2_**C**H_3_). Elemental Analysis for C_43_H_54_O_9_ (714.88): calc. C 72.24, H 7.61; found C 73.47, H 7.71.

*4′-({4′′-[(Octyloxy)carbonyl]phenoxy}carbonyl)phenyl undec-10-en-1-yl terephthalate* (UTHH8)

A mixture of benzoic acid **7** (4.0 g, 7.71 mmol) and undec-10-en-1-ol (1.33 g, 7.65 mmol) was dissolved in dichloromethane (100 mL) and cooled to 2–8 °C. Dicyclohexylcarbodiimide (DCC) (1.97 g, 9.36 mmol) and 4-(*N,N*-dimethylamino)pyridine (DMAP) (0.19 g, 1.56 mmol) were added, and the mixture was stirred for two hours, during which it was warmed to room temperature. Precipitated dicyclohexylurea was filtered off, and the resulting filtrate was diluted with dichloromethane (100 mL), washed with HCl (20 mL, 1:15) and water. The organic layer was dried with anhydrous sodium sulphate. The solvent was removed under the reduced pressure and the crude product was purified by column chromatography on silica (eluent dichloromethane:acetone, 99.8:0.2) Further re-crystallisation from hexane yielded 4.77 g (93%) of UTHH8 final product.

^1^H NMR (CDCl_3_) 8.25–8.37 (4 H, m, H-2, H-6, H-3′, H-5′), 8.10–8.25 (4 H, m, H-3, H-5, H-3′′, H-5′′), 7.43 (2 H, d, *J =* 8.8, H-2′, H-6′), 7.32 (2 H, d, *J =* 8.2, H-2′′, H-6′′), 5.71–5.93 (2H, m, C**H**_2_=CH), 4.89–5.07 (1H, m, C**H**=CH_2_), 4.29–4.44 (4 H, m, COOCH_2_, OCH_2_), 2.05 (2 H, q, *J =* 6.8 Hz), 1.79–1.92 (4 H, m, 2 × C**H**_2_CH_2_O), 1.22–1.54 (22 H, m, 11 × CH_2_), 0.90 (3 H, *J* = 6.5, C**H**_3_CH_2_). ^13^C NMR (75 MHz, CDCl_3_): 165.63 (2 × COOCH_2_), 163.82 (2 × COOAr), 155.07 (C-1′), 154.35 (C-1′′), 139.18 (**C**H=CH_2_), 135.22 (C-4),132.54 (C-1), 132.00 (C-3′, C-5′), 131.21 (C-3′′, C-5′′), 130.21 (C-2, C-6), 129.77 (C-3, C-5), 128.26 (C-4′′), 126.91 (C-4′), 122.04 (C-2′, C-6′), 121.68 (C-2′′, C-6′′), 114.14 (CH=**C**H_2_), 65.79 (COO**C**H_2_), 65.32 (COO**C**H_2_), 33.79 (**C**H_2_CH=CH_2_), 31.79 (**C**H_2_CH_2_CH_3_), 29.01–29.72 (m), 28.90 (7 × CH_2_), 28.71 (**C**H_2_CH_2_O), 28.62 (**C**H_2_CH_2_O), 26.03 (**C**H_2_(CH_2_)_2_O), 26.00 (**C**H_2_(CH_2_)_2_O), 22.64 (**C**H_2_CH_3_), 14.10 (CH_2_**C**H_3_). Elemental Analysis for C_41_H_50_O_8_ (670.83): calc. C 72.24, H 7.61; found C 73.22, H 7.67.

### 2.2. Experimental Methods and Techniques

The sequence of mesophases was determined by the observation of the characteristic textures and their changes in a polarising optical microscope (POM), Nikon Eclipse E600POL (Nikon, Tokyo, Japan). Planar cells (bookshelf geometry) of 12 μm thickness (glasses with indium tin oxide transparent electrodes (5 × 5 mm^2^) were supplied by Military University of Technology (Warsaw, Poland)). The cells were filled with the studied material in the isotropic phase by means of capillary action. The texture observation on the samples with homeotropic alignment, e.g., free-standing films (FSF), was also performed; while preparing the FSF, the liquid crystalline material was mechanically spread over a circular hole (diameter 3 mm) in a metal plate placed in the hot stage. The heating/cooling stage Linkam LTS E350 (Linkam, Tadworth, UK) with a TMS 93 temperature programmer was used for the temperature control, which allows temperature stabilisation within ±0.1 K.

The phase transition temperatures were determined by differential scanning calorimetry (DSC) using a Perkin–Elmer DSC8000 calorimeter (PerkinElmer, Shelton, CT, USA). The samples of about 4–8 mg, hermetically sealed in aluminium pans, were placed into the calorimeter chamber filled with nitrogen. The calorimetric measurements were performed on cooling/heating runs at a rate of 5 K min^−1^ for the precise evaluation of the phase transition temperatures. The kinetics of the phase transition temperatures was studied with heating/cooling rates of 1, 2, 3, 5, 10, 20, 30, 40 and 50 K min^−1^. The temperature and enthalpy change values were calibrated on the extrapolated onset temperatures and enthalpy changes of the melting points of water, indium and zinc.

X-ray diffraction (XRD) measurements were performed to determine the structural properties of the smectic mesophases. Experiments in the small diffraction angle range allowed the determination of the smectic layer spacing, d. A Bruker D8 Discover system was used (parallel beam of CuK_α_ radiation, λ = 1.54 Å, formed by Goebel mirror, Anton Paar DCS 350 heating stage, scintillation detector), and the temperature stability was 0.1 K. Samples were prepared in the form of thin films on a heated surface. The smectic layer thickness was determined using Bragg’s law: nλ = 2d sinθ, where *n* is a positive integer and θ is the angular position of the diffraction peak.

## 3. Results and Discussion

This section contains the experimental results obtained on several reactive mesogens with different molecular structure by POM, DSC and XRD techniques, together with the related discussion of the obtained results in terms of the molecular structure–mesomorphic property relationship.

### 3.1. Mesomorphic Behaviour

**Figure 3:**
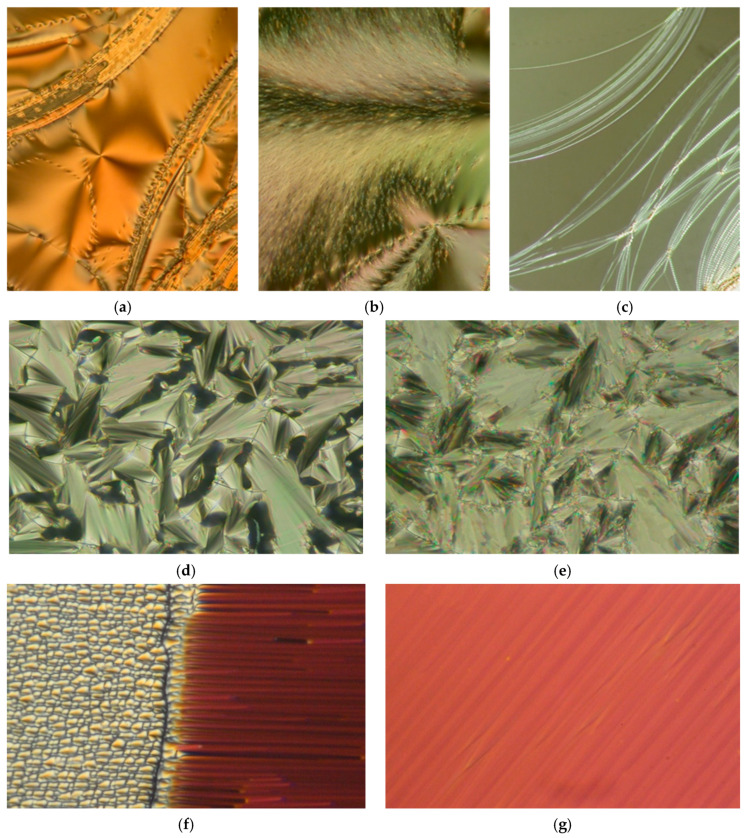
Microphotographs of the reactive mesogens’ characteristic textures obtained by cooling in POM: (**a**) FSF of the SmC phase for UTHH8 at 105 °C, (**b**) FSF of the SmC-Cr phase transition for UTHH8 at 66 °C, (**c**) FSF of the SmA-SmC phase transition for UTHH8, (**d**) planar fan-shaped texture of the Iso-SmA phase transition for UTHH8 at 138 °C, (**e**) planar texture (broken fans) of the SmC phase for UTHH8 at 107 °C, (**f**) planar texture of the N-SmA phase transition for UVHG at 92 °C, (**g**) well-aligned planar texture of the SmA phase for UVHG at 120 °C. Width of the photos is about 300 μm.

For the newly designed reactive mesogens, the sequences of mesophases were determined from the characteristic textures and their changes observed in a polarising optical microscope. The representative textures obtained by POM on planar samples and free-standing films are presented in Figure 3.

**Figure 4:**
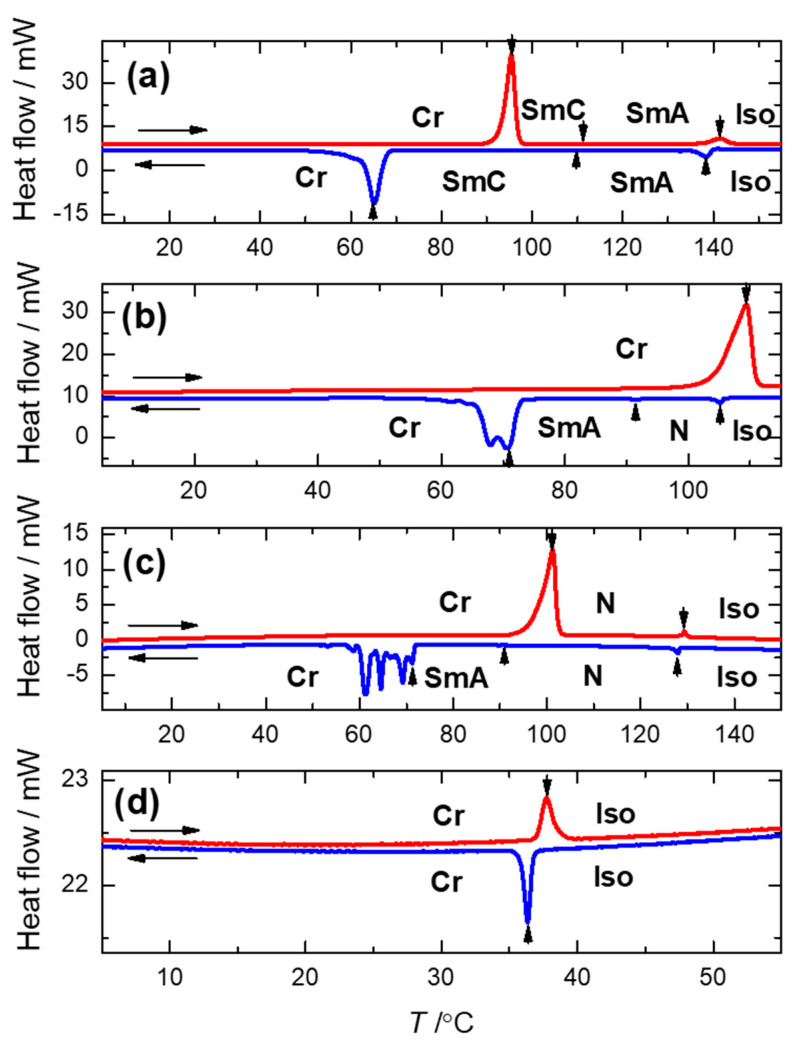
DSC plots of the heating/cooling runs (red/blue curves, respectively) for: UTHH8 (**a**), UVHG (**b**), UKHG (**c**) and UKHM (**d**) ester-based materials. The vertical arrows indicate the peaks corresponding to phase transitions, and the mesophases are indicated.

The phase transition temperatures and transition enthalpies were evaluated from DSC measurements, and the results are summarised in Table 1. The DSC plots of the second heating/cooling runs for the selected reactive mesogens are shown in Figure 4.

For the UKHM mesogen with the methyl group as a lateral substituent and a branched terminal chain which is sterically unfavourable to create mesophases, the liquid crystalline behaviour was not detected. Nevertheless, it can potentially be utilised as a specific co-monomer for the design of the complex macromolecular materials. All the other mesogens clearly possess the liquid crystalline behaviour over a reasonably broad temperature range. The richest mesomorphic behaviour was detected for the UKHG reactive mesogen with the methyl lateral substituent in Y-position (see Figure 1). The nematic, the SmA and the SmC phases were clearly found upon cooling from the isotropic (Iso) phase. The non-substituted UTHH8 reactive mesogen exclusively possesses the smectic phases, namely the orthogonal SmA and the tilted SmC phases. Figure 3a–c shows the homeotropic textures of the detected smectic mesophases for the UTHH8 reactive mesogen obtained on the FSF.

It can be concluded that the lateral substitution placed at the Y-position on the specific molecular core (see Figure 1), in combination with the appropriate length of the chain far from the polymerisable vinyl group, makes the nematic phase favourable upon cooling from the isotropic phase. This is fully confirmed by two UVHG and UVHGET reactive mesogens which possess the nematic and the SmA phase upon cooling, while the tilted SmC phase was not detected for those two materials. While comparing those two mesogens with the lateral methoxy group, the shorter chain placed far from the polymerisable vinyl group results in an extension of the SmA phase temperature range upon cooling, while the melting point is found almost unaffected. The texture of the N-SmA phase transition for UVHG mesogen obtained on the planar sample is presented in Figure 3f. It was easy to obtain a very homogeneous alignment in the SmA phase for the UVHG reactive mesogen (see Figure 3g). The textures observed on the planar samples are presented in Figure 3d,e for the fan-shaped SmA phase and for the broken fan-shaped SmC phases. The tilted SmC phase was found to be fully monotropic (i.e., overcooled as it appears upon cooling only) for both UKHG and UTHH8 reactive mesogens. However, it can be expected that under definite conditions, the tilted SmC phase can be stabilised and even extended for the foreseen respective macromolecular materials after the polymerisation.

**Figure 5:**
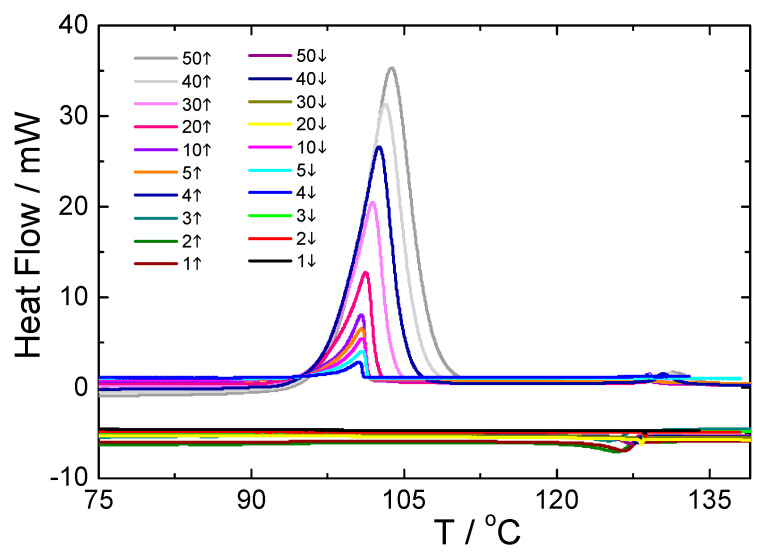
Heating and cooling DSC runs for the UKHG reactive mesogen measured at different heating/cooling rates (K · min^−1^), as indicated.

For the UKHG reactive mesogen, the kinetics of the phase transitions was checked using DSC measurements under several cooling rates, as presented in Figure 5, where a clear situation regarding the behaviour of the melting point is shown.

**Figure 6:**
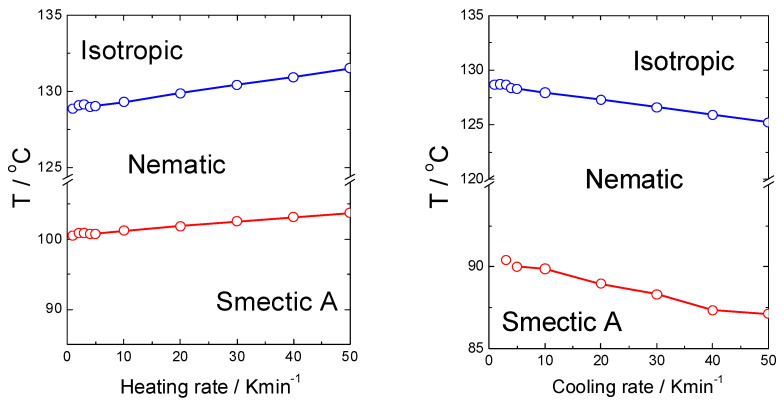
Resulting phase diagrams for the UKHG reactive mesogen obtained upon heating (**left**) and cooling (**right**) runs, indicating a clear difference of the phase transition temperatures obtained upon heating/cooling DSC measurement rate.

For this study, only the data from the second cooling runs were used. It has been found that the stability of all the mesophases slightly depends on the rate of cooling, which is quite obvious. While increasing the cooling rate, the Iso-N and N-SmA phase transition temperatures slightly decreased, and the stability of the phases increased, upon heating, the situation was the opposite (see Figure 6). However, the transition to the solid crystal (Cr) phase shows a clear sign of super-cooling [34,51], being more pronounced especially at cooling rates higher than 40 K. This effect is also related to the monotropic character of the lower temperature range of the SmA phase.

### 3.2. Structural Properties

**Figure 7:**
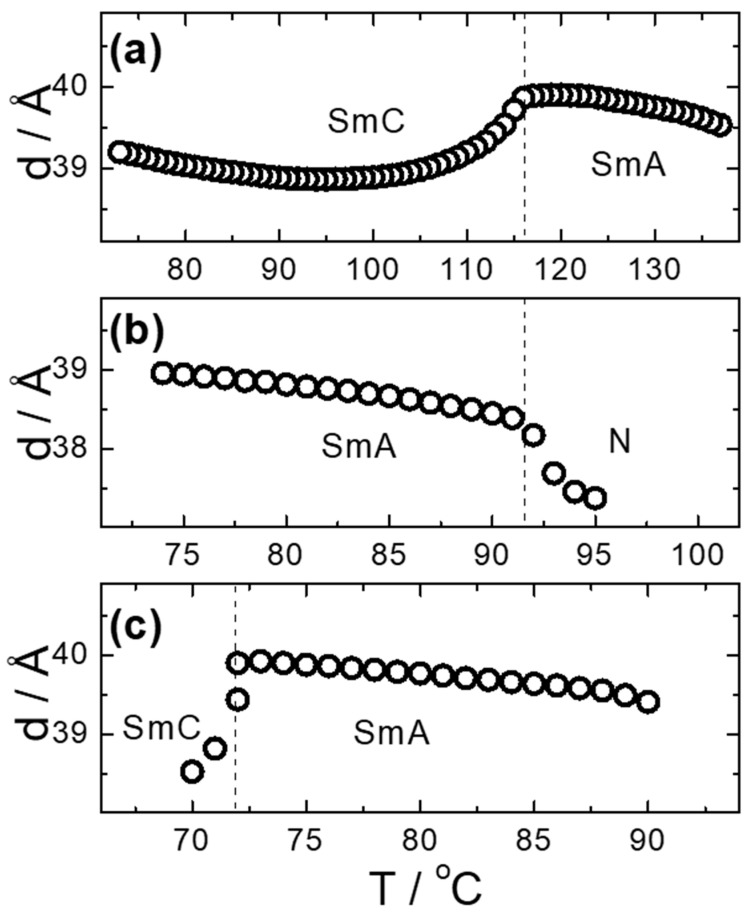
Temperature dependence of the smectic layer spacing, d(T), for the selected reactive mesogens: (**a**) UTHH8, (**b**) UVHG and (**c**) UKHG. Dashed lines indicate the phase transitions, and the mesophases are designated. In nematic phase, the measured d values are related to the short-range positional order, as evidenced by a considerable broadening of the related diffraction signals.

For three reactive mesogens exhibiting the smectic phases, the small-angle X-ray scattering measurements were performed in order to confirm the mesophase identification and to determine the smectic layer spacing. The results, namely the temperature dependence of the layer spacing, d, for UTHH8 (a), UVHG (b) and UKHG (c) reactive mesogens are presented in Figure 7. All studied materials exhibit a slight increase of d values in the SmA phase upon cooling, which results from the growing orientational order of the molecules and increasing number of all-trans molecular conformers. At the SmA-SmC phase transition, a typical drop in d values appears, caused by the tilting of the molecules with respect to the smectic layer normal in the SmC phase. Upon further cooling towards crystallisation, the tilt angle saturates and the layer spacing starts to increase due to the same factors as described above for the SmA phase (see Figure 7a for UTHH8 reactive mesogen).

For the calculation of the length of the reactive mesogen molecules in the energy-optimised conformation, the MOPAC/AM1 model was used. The resulting molecular structures with the principal axis of minimum moment of inertia, e.g., the long molecular axis, are shown in Table 2. Taking into account the most extended conformer, the length of the molecule, L, is found to be slightly higher than 42 Å for four of the studied materials; naturally, molecules of the UVHGET reactive mesogen with the short terminal chain are considerably shorter, L = 37.7 Å, than that for the other materials. For three reactive mesogens exhibiting the orthogonal SmA phase, namely UTHH8, UVHG and UKHG, the layer spacing was found to be slightly lower than the lengths of the respective most extended conformers, which can be explained by the non-perfect orientational order of the molecules in the smectic layers. We compared the non-substituted UTHH8 reactive mesogen with its substituted analogues, UVHG and UKHG, having the bulky methoxy and methyl lateral substituents, respectively. From the comparison, it can be concluded that the lateral substitution on the molecular core close to the alkyl chain with polymerisable vinyl group has only a minor impact on the resulting molecular shape. This might be because the layer spacing in the orthogonal SmA phase remains almost the same for those three reactive mesogens [40].

The orientational order parameter, S2, can be estimated according to the relation d **≈** L/3(S2 + 2) [40,52], where d is the layer spacing in the orthogonal SmA phase and L is the length of the most extended conformer. With actual values for UTHH8 (L = 42.2 Å; d = 39.8 Å), for UKHG (L = 42.6 Å; d = 39.7 Å) and for UVHG (L = 42.4 Å; d = 38.5 Å), S2 = 0.83 (UTHH8), S2 = 0.80 (UKHG) and S2 = 0.72 (UVHG) can be obtained, which are quite typical values of S2 orientational order parameter in fluid smectics. It can be summarised that the orientational order parameter S2 is decreased with the increasing size of the lateral substituent.

## 4. Summary of the Results and Conclusions

Several new calamitic reactive mesogens, with vinyl terminal group with different length of flexible chains and with/without lateral substitution by the methyl and methoxy groups placed on the molecular core, have been designed and synthesised. Depending on the molecular structure, the reactive mesogens exhibit the nematic, orthogonal SmA and tilted SmC phases in a reasonably broad temperature range. The structure of the smectic mesophases has been confirmed by XRD measurements. The calculated length of the most extended conformer correlates well with the smectic layer spacing obtained experimentally. Lateral substitution by a bulky methoxy group on the molecule core deteriorated the arrangement of the molecules (UVHG and UVHGET), and only nematic and orthogonal SmA phases were detected. The lateral substitution by the methyl group on the molecular core had a very positive effect on the self-assembling behaviour (UKHG), and rich mesomorphic behaviour (N-SmA-SmC) was observed. While comparing the mesomorphic behaviour for UKHG and UVHG reactive mesogens differing by the substituent type only (i.e., CH_3_ and CH_3_O, respectively), it is possible to conclude that the presence of a bulky methoxy group makes the tilted smectic phase unfavourable. However, a lateral methyl substitution in combination with a branched terminal chain (UKHM) fully suppressed the self-assembling behaviour of the resulting material with a given type of molecular core. The kinetics of the phase transitions for UKHG reactive mesogen has been studied in dependence on the heating/cooling rate of DSC runs. The resulting phase diagrams for the UKHG reactive mesogen clearly demonstrated a noticeable difference in the phase transition temperatures on the heating/cooling DSC rate. The Iso-N and N-SmA phase transition temperatures slightly decreased while increasing the cooling rate.

The reactive mesogens reported here with a functional vinyl terminal group can be further used as reactive mesogens, i.e., both the monomers and co-monomers, for the design of the smart self-assembling macromolecular materials, such as siloxane-based polymers, co-polymers and elastomers, exhibiting self-assembling behaviour favourable for various applications, including smart sensors [4,52,53,54,55] as well as tunable liquid crystal micro-lens array [56], dynamic focusing micro-lens array [57] and spatial modulators for working in THz aimed for photonics and telecommunications systems [58].

## Figures and Tables

**Table 1:** Sequence of phases (PH) determined by POM, melting points (m.p.) and clearing points (c.p.) (°C) measured upon heating, phase transition temperatures (°C) measured upon cooling (10 K min^−1^) and respective enthalpy values ΔH (J · g^−1^) determined by DSC for the reactive mesogens. Symbol “**-**” represents if the phase does not exist.

Mesogen	m.p.	c.p.	pH	T/ΔH	PH	T/ΔH	PH	T/ΔH	pH	T/ΔH	pH
UKHG	101.3 (+72.2)	129.4 (+0.9)	Cr	67.8 (−63.2)	SmC	73.0 (−0.02)	SmA	90.5 (–0.02)	N	128.7 (−1.1)	Iso
UKHM	37.7 (+1.8)	37.7 (+1.8)	Cr	36.3 (−2.2)	-		-		-		Iso
UVHG	109.4 (+83.2)	109.4 (+83.2)	Cr	70.5 (−61.9)	-		SmA	91.7 (–0.4)	N	105.1 (−1.3)	Iso
UVHGET	103.5 (+60.1)	125.1 (+0.9)	Cr	31.5 (−17.5)	-		SmA	91.9 (–0.1)	N	124.2 (−1.0)	Iso
UTHH8	95.4 (+90.2)	141.5 (+8.0)	Cr	65.1 (−75.2)	SmC	113.7 (−0.1)	SmA	138.4 (–8.4)	-		Iso

**Table 2:** Conformation of the reactive mesogen molecules after energy minimisation using MOPAC/AM1. L is the length of the most extended conformer.

Mesogen Short Name and the Cartoon of the Most Extended Molecule	L (Å)
**UVHG** 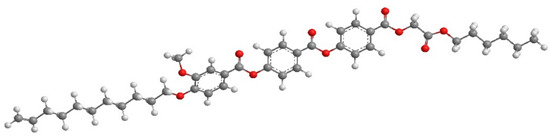	42.4 Å
**UKHG** 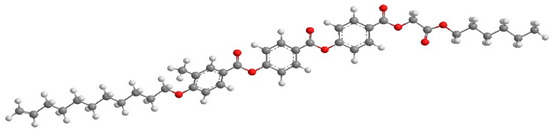	42.6 Å
**UKHM** 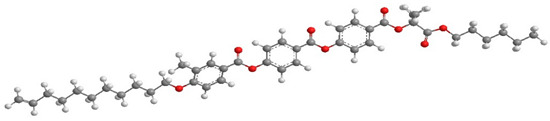	42.6 Å
**UVHGET** 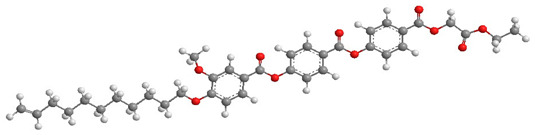	37.7 Å
**UTHH8** 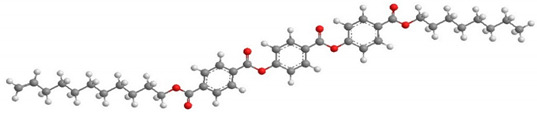	42.2 Å
